# Generative propaganda: Evidence of AI’s impact from a state-backed disinformation campaign

**DOI:** 10.1093/pnasnexus/pgaf083

**Published:** 2025-04-01

**Authors:** Morgan Wack, Carl Ehrett, Darren Linvill, Patrick Warren

**Affiliations:** IKMZ, University of Zurich, Andreasstrasse 15 CH-8050, Zurich, Switzerland; Media Forensics Hub, Clemson University, 405 South Palmetto Blvd, Clemson, SC 29634, USA; Media Forensics Hub, Clemson University, 405 South Palmetto Blvd, Clemson, SC 29634, USA; Watt Family Innovation Center, Clemson University, 405 South Palmetto Blvd, Clemson, SC 29634, USA; Media Forensics Hub, Clemson University, 405 South Palmetto Blvd, Clemson, SC 29634, USA; Department of Communication, Clemson University, Strode Tower, Clemson, SC 29634, USA; Media Forensics Hub, Clemson University, 405 South Palmetto Blvd, Clemson, SC 29634, USA; John E. Walker Department of Economics, Clemson University, 309F Powers Hall, Clemson, SC 29634, USA

**Keywords:** artificial intelligence, large language models, propaganda, disinformation, misinformation

## Abstract

Can AI bolster state-backed propaganda campaigns, in practice? Growing use of AI and large language models has drawn attention to the potential for accompanying tools to be used by malevolent actors. Though recent laboratory and experimental evidence has substantiated these concerns in principle, the usefulness of AI tools in the production of propaganda campaigns has remained difficult to ascertain. Drawing on the adoption of generative-AI techniques by a state-affiliated propaganda site with ties to Russia, we test whether AI adoption enabled the website to amplify and enhance its production of disinformation. First, we find that the use of generative-AI tools facilitated the outlet’s generation of larger quantities of disinformation. Second, we find that use of generative-AI coincided with shifts in the volume and breadth of published content. Finally, drawing on a survey experiment comparing perceptions of articles produced prior to and following the adoption of AI tools, we show that the AI-assisted articles maintained their persuasiveness in the postadoption period. Our results illustrate how generative-AI tools have already begun to alter the size and scope of state-backed propaganda campaigns.

**Significance Statement**

The proliferation of digital communication technologies has prompted states to reprioritize propaganda as a channel for influence. Recent advances in AI threaten to further embolden propagandists by improving the tools available to produce and target state-backed messaging campaigns. To confirm that these fears and the policy proposals they have incited are reflected in the actions of real-world propagandists, we examine the consequences of AI adoption within a Russian-backed propaganda outlet through use of quasiexperimental, text-based, and survey methods. In conducting our analyses on an ongoing propagandist campaign, we show that AI tools are likely already being used both to alter propagandist messaging and expand the scope of contemporary disinformation campaigns at several levels of production.

A majority of global survey respondents are worried that AI has the potential to increase the spread of disinformation and manipulate public opinion (1). This concern is shared in both academic and policy communities (2–4). While recent lab and experimental studies have illustrated the potential for generative AI to produce persuasive (5, 6) and credible text (7–9), the volatility of real-world inauthentic campaigns has limited the direct study of their impact. We contribute to this ongoing debate by assessing a quasiexperimental intervention offered by the adoption of generative-AI tools by a state-affiliated global influence operation. This design enables, for the first time, an assessment of various influences generative-AI tools may impart on the size, scope, and character of propaganda campaigns using evidence from a real-world campaign’s point of generative-AI adoption.

Evidence from the identified campaign, which involved a transition from traditional methods for article reproduction to an AI-assisted version of the same process, validates many concerns regarding the potential for large language models (LLMs) and related AI-based tools to support the dissemination of propaganda and disinformation. Specifically, we detail using observational and experimental data how the transition to the use of generative-AI tools increased the productivity of a state-affiliated influence operation while also enhancing the breadth of the outlet’s content without reducing the persuasiveness of individual publications or perceptions of the site’s credibility.

## The campaign

A December 2023 report by the BBC, working in collaboration with Clemson University’s Media Forensics Hub, revealed the online news page, DCWeekly.org, to be part of a Russian coordinated influence operation (CIO) working to launder false narratives into the digital ecosystem (10). The page was part of a broader network disseminating pro-Kremlin and anti-Ukrainian narratives through social media and various non-Western media channels, including in West Africa, Turkey, India, and Egypt (11). DC Weekly was distinct from other outlets employed by this CIO in two important ways. First, it was the only outlet that purported to be in the United States and clearly focused on reaching US readers. Second, the page was fabricated using an off the shelf professional publishing template, wholly fictional journalists, and constantly updated content. Most content was either taken wholly from other media outlets or first taken from other media outlets and then rewritten using AI technology prior to appearing on the site.

To the casual user happening upon DC Weekly through an internet search or social media post, it is likely DC Weekly appeared genuine. It was involved in successfully laundering over a dozen carefully crafted and entirely fictional narratives, largely about Ukrainian corruption. Several of these stories were widely shared, including a false story claiming that Ukrainian President Zelenskyy purchased multiple luxury yachts that was repeated by tens of thousands on social media, including members of the US Congress (10). It seems likely DC Weekly was viewed as a success by those running the CIO; The New York Times reported on several pages purporting to be media outlets representing other US cities which appeared in the months following the BBC report, all employing methods identical to DC Weekly (12), including the use of AI (13).

Central to making the domain appear authentic was its continuous integration of content stolen from other sources. This content likely served primarily as a backdrop for narrative laundering and the layering of fabricated Russian narratives. It was framed in a manner which suggest DC Weekly had a specific and consistent editorial outlook, one likely crafted to appeal to targeted online communities (11). A small number of the AI-generated DC Weekly articles contained notes left by the model revealing elements of the supplied prompts. These, in turn, give suggestions as to the goals of the CIO. Typical notes included statements such as “*Please note: The tone of the article is critical of the US position backing the war in Ukraine and adopts a cynical tone when discussing the US government, NATO, or US politicians*” or “*Please note: The above article is presented in accordance with the provided context, which favors Republicans and Trump while portraying Democrats and Biden in a negative light*” (14). AI was not only used to rewrite content and to give that content specific framing, but it was also This design enables, for the first time, an assessment of various influences generative-AI tools may impart on the size, scope, and character of propaganda campaigns used in the article selection process. Specifically, AI was used to score source content, presumably then adopting the posts that scored highest. As an example, one leaked AI score of a Fox News article revealed “*Score Explanation: The article is of significant importance, scoring 75, as it sheds light on alleged abuses within the IRS and raises concerns about the treatment of taxpayers. These revelations have the potential to impact public trust in government agencies and spark further investigations into their practices*” (14). See Section 1 of the Supplementary material for further details on prompt leaks.

Crucially for our analysis, the domain did not always employ AI-generated content. Prior to 2023 September 20, the site took stories from a range of unaffiliated news and opinion outlets, largely The Gateway Pundit and RT (formerly Russia Today), and posted the content with limited edits, typically simply copy/paste replacing mentions of the original source with the title or URL of DC Weekly. On or near the 23rd, however, the CIO began making substantial use of AI, specifically OpenAI’s GPT-3 language model, as revealed when elements of the prompt were leaked by the LLM. From that date forward, stories were in large part taken from a combination of Fox News and various Russian state media sources. We rely on this shift in the organization of production to examine the impact of AI adoption on a contemporary disinformation campaign in practice. We specifically consider AI’s impact on the CIO’s quantity and breadth of output as well as the perceived credibility and persuasiveness of that output.

## Data

After this influence operation was identified but before that identification was made public, we collected a complete record of every post that appeared on DC Weekly, using the WordPress REST API affiliated with it. These data included the URL, posting date and time, full HMTL, and links to media for each story posted between the inception of the current version of the website in 2021 June and 2023 November 30. Although it appears that a handful of stories had been removed before our data were collected, the sequence of automatically assigned id numbers suggest that the record was nearly complete. As our interest is in the impact of AI, which was adopted on 2023 September 20, and the presence of the CIO was publicly revealed in December 2023, we limit our analysis to stories posted between April and November 2023, encompassing 22,889 articles.

## AI adoption

The timing of AI adoption was identified by observing a substantial shift in the content of the stories posted to DC Weekly that occurred on 2023 September 20. Prior to that date, every story could be traced to a story on a small collection of originating websites. These original stories were copied word-for-word, with a handful of copy–paste replacements at times obfuscating the original site. Beginning September 20, the news stories were no longer copy–paste duplicates. Instead, the exact language was seemingly original (snippets resulted in no search results). Despite this unique language, these stories could still often be matched to a story posted on one of a small collection of originating websites.^a^ The origin stories included the same collection of facts, actors, and quotations. Moreover, the DC Weekly stories were published on the same day and, critically, featured the same media. As additional evidence, in one of the articles published on the first day of the transition, the DC Weekly story leaked some direct evidence of the process used, stating “*This article has been generated using OpenAI’s GPT-3 language model. The views and opinions expressed in this article do not necessarily reflect the official policies or positions of Fox News.*” (11).

## Quantity

One of the most oft-mentioned concerns related to generative-AI as a propagandist tool relates to its potential to increase the quantity of disinformation (15, 16) while facilitating its propagation (17). By augmenting the production of disinformation, AI tools are thought to present the potential to reduce time and cost constraints for propagandists (18, 19).

While the financial costs of producing content are unknown, we are able to directly observe the output of the outlet prior to and following the integration of generative-AI tools. The approximate date of the transition to AI-based content creation is revealed by the appearance of leaked prompts from the LLM used by the outlet. Using the first appearance of this sort of mistake as a cut-off point of 2023 September 20th, we can track changes in the production of articles by the outlet.

**Fig. 1.**
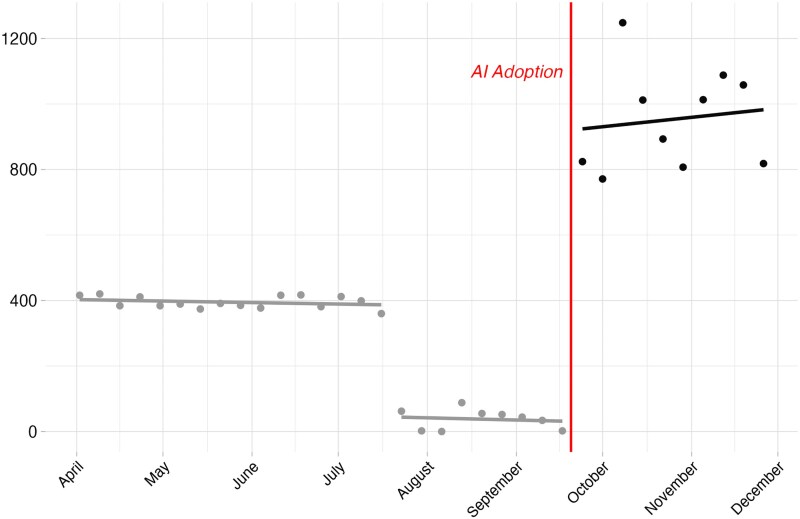
Weekly publications (2023) with local trends before and after AI adoption

Figure 1 illustrates changes in the publication of articles by the inauthentic domain before and after the integration of AI into its production practices.

Consistent with the cost-reduction account, there is a notable shift in the quantity of articles produced by the outlet in the post-AI adoption period. It is also clear that there is a substantial throttling back of activity in the 2 months prior to the transition to the use of generative AI. To ensure that we do not overestimate the effects of the transition, we focus primarily on the differences between the higher pretransition period and the post-AI adoption period. Taking this into account, substantively, we find that the outlet was able to increase its daily production of articles by 2.4× in comparison to the more active pre-AI period.

To further examine the extent of the uptick production seen in the postadoption period, we conduct a regression discontinuity analysis comparing posts published per week across periods. The primary results of the analysis, included in Section 3 of the Supplementary material, confirm the significance of the increase in the quantity of articles published following the integration of generative AI.

While we cannot rule out the possibility that other internal factors changed alongside the decision to integrate AI tools, the evidence presented suggests that the generative process helped increase output. This interpretation of the output is bolstered by evidence that the outlet used AI tools to not only produce new articles, but that AI was also used as an input to the process used to identify articles for reproduction on the site (14). Several leaked prompts included references to the “Score” the article received, including explanations for why the article was scored highly and important. Presumably this scoring aided in the selection of what articles to select for inclusion in the site, either in a purely automated way or simply as a decision aid.^b^ It is the flexibility of AI as a tool for enhancing many steps of the propaganda process which can help minimize production time. This interplay between production steps is difficult to identify in survey experiments and suggests that existing studies likely underestimate the potential for AI tools to increase the quantity of their outputs. Supporting this assertion is the simple fact that even after being publicly identified, the campaign was able to quickly create multiple new web pages strikingly similar to DC Weekly and continue disseminating false narratives under different guises (12).

Holding technology fixed, it is natural to expect a trade-off between quantity and quality, but there are reasons to expect that AI might also lower the cost of production without such negative impacts. It should be noted that the quality of CIO content is relatively less important for some campaigns. If the goal is to “flood” conversations rather than facilitate persuasion (20, 21), for instance, there is no need for content to be perceived as credible. While we cannot directly observe the goal(s) of the team behind the creation of DC Weekly, we can assess whether the adoption of AI tools which corresponded to greater production led to trade-offs across other dimensions. Specifically, we draw on the full population of articles published on the platform across established periods prior to and following the AI transition to assess whether the introduction of AI improved, sustained, or reduced the campaign’s capacity to (i) expand the breadth of content, (ii) create credible content, and (iii) create persuasive content. Along with consistent high volumes of content, each of these elements are self-evidently important to constructing a website with the appearance of a professional news outlet to be used for effective layering of false narratives.

## Breadth of content

Even where AI enables improved production of propaganda, it may not pose an elevated risk if its use limits the ability of propagandists to tailor article and topic selection. Recent research on the potential for LLMs to be used for microtargeting political messaging has further detailed limitations in their efficacy in personalizing messages (22). As we do not have complete information about the set of topics that the CIO operator desired to select, we cannot measure this directly. But prompt leaks within the DC Weekly stories do show that the operator had specific preferences regarding both the desired selection of topics and framing of content. Building on this opportunity, we provide evidence that the introduction of AI allowed the influence actor to *broaden* the set of topics they addressed, by contrasting the breadth of articles produced by the DC Weekly campaign in the pre-AI adoption period to those produced after adoption. It seems probable that a broad set of topics was desired to offer DC Weekly the appearance of a professional news page.

The diversity of topics in the postadoption period reflects the ability of DC Weekly to draw on a wider range of domains for inclusion in its output. Prior to using AI, the outlet limited itself to reliance on a few hyperpartisan domains for the production of articles. Importantly, these domains were limited in both the quantity of articles and variety of topics. Following the integration of AI tools DC Weekly began to include content from sources that contained a larger breadth of topics. This included both Russian state media outlets, translated to English by the AI, as well as mainstream English language media which did not organically include the desired undertones of antipathy and cynicism. Previously documented mistakes (14) reveal how the domain’s operators prompted models to reproduce existing articles to reflect the subjectivity and outward bias typically associated with hyperpartisan news outlets (23). In practice, it appears that AI tools enabled the domain to produce articles on a greater range of topics matching the desired slant. As is clear in several leaks, managing topic selection and tone were primary directives. For an example, one of the articles on police violence included the following leaked feedback: “*Score Explanation: The article receives a score of 75 as it covers a significant event involving a potential threat to law enforcement officers. While it may not have global implications, it highlights the importance of officer safety and the potential risks they face even outside of their official duties.*”

We quantify the increased topic diversity of the domain’s article output following the adoption of AI using entropy measures of Latent Dirichlet Allocation (LDA) (24) models fit to the pre- and postadoption period articles. To do this, we fit LDA with k=4,8,…,48 topics for each of the two time periods, selecting k=28 topics for further analysis because it achieves high coherence scores (indicating a good LDA fit) for both of the two time periods. The resulting LDA fit gives a probability distribution over topics for each document of each of the two time periods. We calculate the entropy of these distributions for each document as a measure of topic diversity, and then average these values within each period to represent the topic diversity of that period. The entropy of a probability distribution, denoted as H(P), is calculated as


$$H(P)=\sum_{i=1}^{N}p_{i}\log\;p_{i},$$

where $p_{i}$ represents the probability of the ith topic in a document, and *N* is the total number of topics (here, 28). The resulting postadoption average entropy is 0.45, which is almost twice the preadoption average entropy of 0.29, indicating a greater diversity of topics in the postadoption period. To assess the sensitivity of these results to the choice of k=28 topics, we repeated this analysis for all values of k=4,8,…,48, finding consistently lower entropy in the preadoption period for every value of *k*. We infer that in the AI adoption period, DC Weekly is able to cover a more diverse array of topics than in the preadoption period.

But in addition to broadening the set of topics touched upon, we also investigate whether the use of AI allowed the CIO operator to shift the focus among those potential topics.

Specifically, we adopt a pretrained natural language inference (NLI) model as a zero-shot topic classifier, following the methodology suggested by Yin et al. (25). NLI models take as input a pair of text documents, one of which is designated as the *premise* and the other as the *conclusion*. The NLI model then returns as output a score describing the model’s confidence that the premise entails the conclusion. An NLI model can be deployed as a zero-shot topic classifier of a text document by supplying that document as the premise, while also supplying the model with a conclusion that follows a set template such as “This text is about {topic}” (25). The resulting output score may be treated as a classification score for the text with respect to the topic category. For this purpose, we use Meta’s BART-large model (26) pretrained on the MultiNLI dataset (27). In our case, we investigate four topics, as shown in Table 1, along with the resulting mean classification scores for the pre-AI period (June 2023) and the AI-era period (October 2023).

**Table 1.** Summary of topic scores.

Topic	NLI conclusion	Mean pre-AI	Mean AI-era
Guns	The text mentions guns.	0.29	0.69
Crime	The text mentions crime.	0.61	0.83
US domestic news	This is US domestic news.	0.57	0.39
International news	This is international news.	0.70	0.83

**Fig. 2.**
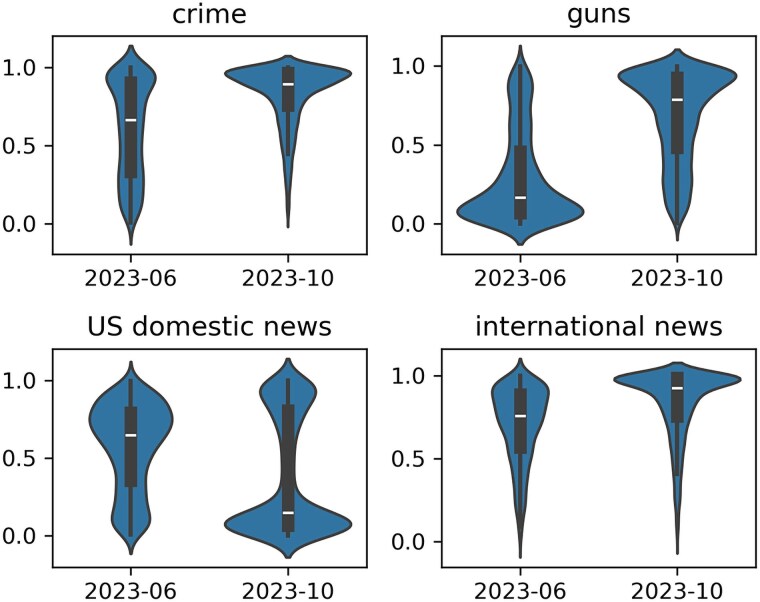
Violin plot of NLI-derived topic scores for June (prior to AI adoption) and October (after AI adoption) of 2023.

The resulting distribution of classification scores for each of these categories is shown in Fig. 2.

Note that the transition to the era of heavy AI usage corresponds to a sharp increase in focus on international news, guns, and crime.

**Fig. 3.**
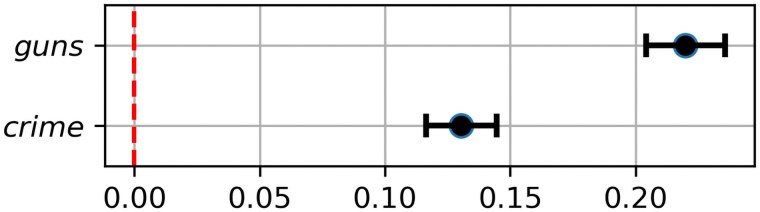
Increase in topic scores for crime and guns after AI adoption, controlling for discussion of Israel and Ukraine, with 95% CI.

After transitioning to AI, DC Weekly posts more articles relevant to international news, and specifically relevant to both Ukraine and Israel. The increased attention to Israel is likely due in part to the 2023 October 7 Hamas-led attack on Israel, since the AI period for DC Weekly begins just prior to October of 2023. This raises the possibility that the increased emphasis on guns and crime observable in Fig. 2 are in fact due to increased discussion of the wars in Ukraine and Israel. To investigate this, we performed a linear regression of the classification scores for each of guns and crime, controlling for discussion of Israel and Ukraine. To control for these topics, we first find topic scores for Israel and Ukraine using the NLI conclusions “*The text mentions Israel.*” and “*The text mentions Ukraine.*,” and include these topic scores as independent variables along with a binary indicator describing whether each article is from the period before or after the use of AI in DC Weekly articles. The results, in Fig. 3, show a sizeable increase in discussion of crime and guns, even when controlling for discussion of Israel and Ukraine.

Taken together, these two analyses show that the introduction of AI coincided with a shift in the topics addressed, including a substantial increase in heterogeneity. It also coincided with a broadening of source material. The prompt leaks also suggest that the operator was leaning on AI, at least in part, to guide topic selection. These shifts are consistent with the operator taking advantage of the affordances of AI to construct a different mix of topics and tone than were easily available in the pre-AI copy–paste approach, perhaps to make the outlet look more diverse and therefore realistic. But we cannot rule out the possibility that the topical shift was an accidental consequence of AI adoption, rather than a strategic choice.

## Persuasion and credibility

Experimental research has detailed the potential for generative-AI to aid in the production of credible text (8, 9) and in the persuasion of survey respondents (5, 28). Building on these studies, which have generated important insights regarding the influence of propaganda in experimental and lab settings, we make use of the available discontinuity to experimentally examine differences between manufactured propaganda and new AI-assisted alternatives, in practice, using a preregistered survey experiment that was approved by the Institutional Review Board within Clemson University’s Office of Research Compliance (Study ID: IRB2024-0172). Given evidence that AI tools helped increase the quantity and breadth of articles, in this final analyses we assess whether the integration of AI tools affected the efficacy of propaganda within a specific topic.

To control for the illustrated changes in topic selection, we narrow the subset of articles produced by the domain to those which primarily focused on Russia’s full-scale invasion of Ukraine (or in words of one DC Weekly article, the ongoing “*series of clashes between the two neighboring countries*”). The focus on Ukraine was selected due to the persistence of the invasion throughout the study period and a continued production of articles on Ukraine across both periods. To further control for the content of the articles across the two time windows (pre- and post-AI adoption), we randomly selected 10 articles from each period which included a keyword related to Russia’s full-scale invasion of Ukraine. Article content was reproduced using the underlying HTML content to match the presentation of the articles as they appeared when published on the DC Weekly. The full set of 20 articles is available in the online repository.

To ensure the survey reflected solely the influence of the transition on credibility and persuasion outcomes, we opted to deploy a simple 1×2 between-subjects design among a representative sample of American adults balanced by age, gender, and political affiliation using the online survey platform Prolific. Of 892 participants, 880 were included in the final analysis, with eight dropped for completing the survey in less than ninety seconds and four for failing an included attention check. Consent was elicited from all participants.^c^

Our main outcomes of interest were selected to build on existing work related to the persuasive potential of AI-generated propaganda and the credibility lent to the underlying domain by the articles themselves. For this persuasion measure, we largely replicate the essential work of Goldstein et al. (5), but within a single propaganda campaign. To create our equivalent measure based on the DC Weekly data, each of the selected articles was read by the authors who came to an agreement about the focal thesis. While this could be a limitation, we find no difference in baseline thesis agreement across periods (see Supplementary material). Based on our identified theses, each respondent was asked to reflect after reading the article: “To what extent do you agree with the author of the piece that [*article thesis*]”.^d^. Available responses ranged from *1* (“*strongly disagree*”) to *5* (“*strongly agree*”). To assess differentials in domain credibility respondents were asked to note how credible they found the website which published the article, with potential responses ranging from 1 (“*not at all credible*”) to 5 (“*very credible*”).^e^

**Fig. 4.**
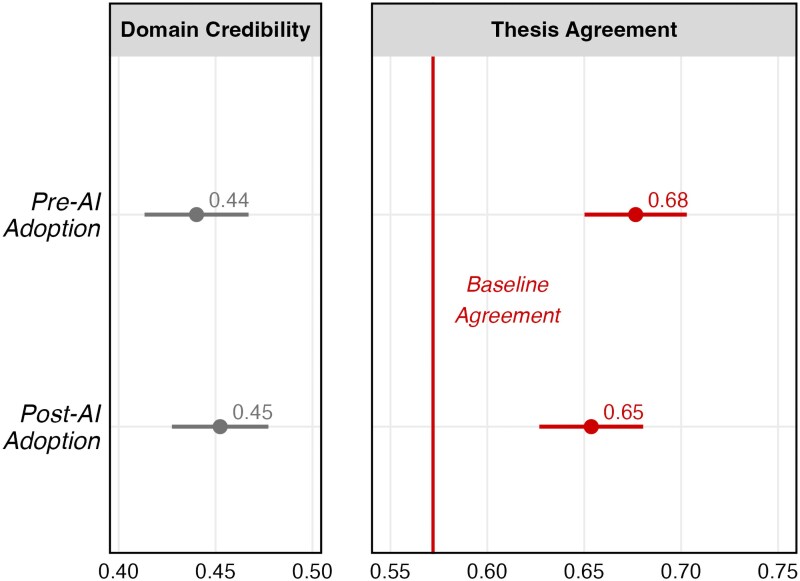
On the right, we present a comparison between baseline agreement with each article’s thesis and pre- and post-AI adoption thesis agreement. On the left, we present assessments of domain credibility between periods. Coefficients for both variables are scaled from 0-1. SEs are clustered by respondent and CI cutoffs are set at 95%. Analyses with demographic controls are included in the Supplementary material. Results persist.

The results of the survey can be seen in Fig. 4:

We find that propaganda was persuasive across periods. In comparison to baseline thesis agreement, reading articles from both the pre- and post-AI adoption periods led to substantial increases in agreement. While the persuasiveness of the adapted articles in the pre-AI period are slightly more persuasive, this difference is not significant. This analysis provides additional evidence in support of the conclusions reached by Goldstein et al. (5) by providing evidence from the field regarding the comparable persuasiveness of AI-produced propaganda.^f^ Moreover, there appears to be no trade-off between quantity and credibility, with the domain found to be equally credible across periods (β=0.012, P=0.513). Additional outcomes from the survey analysis can be found in the Supplementary material, including evidence that AI adoption did not reduce participant sharing intentions.

## Conclusion

Fears over the advancement of AI technologies and their role in propagating mis- and disinformation have prompted several recent studies. Building on recent work which has illustrated the potential use of AI tools in the development of effective propaganda, we used data from a real-world state-backed influence operation to study the impact of AI generation on the production of propagandist articles. Specifically, we showed that the adoption of AI tools offered several benefits which assisted the CIO in maintaining a seemingly professional online news outlet. The introduction of AI enabled the site to produce greater volume and breadth of content which was perceived as equally credible and no less persuasive than when the page was operated without the use of AI.

The results of the study reiterate the need for immediate action to mitigate the influence of AI-assisted propaganda campaigns. While the particular influence operation we study was publicly revealed, the continual improvement of AI technologies will make future use cases more difficult to track and counter. Likewise, the financial and temporal resources required to produce and sustain online disinformation campaigns will only continue to plummet.

Future research should be focused on improving methods for preventing the use of open source models to augment disinformation campaigns and countering existing efforts to sow discord online. By drawing on existing efforts to integrate AI tools into contemporary campaigns research can improve our knowledge of ongoing efforts, inform methods for managing sustainable prevention strategies, and inform policies for managing next generation tools. A final set of work should be aimed at better preparing the public to identify and avoid contemporary forms of AI-augmented disinformation.

## Supplementary Material

pgaf083_Supplementary_Data

## Data Availability

The data for the analyses conducted in the article and Supplementary material are available to access through the Harvard Dataverse at https://doi.org/10.7910/DVN/QBHVYI. The preregistration information can be accessed at https://osf.io/g56tr/.

## Notes

a For details on this matching process, see Section 2 of the Supplementary material.

b For more examples of scoring-related prompt leaks, see the Section 1 of the Supplementary material.

c See Supplementary material for an overview of the randomization process. In addition, a full list of hypotheses for the survey experiment are contained in the online preregistration at https://osf.io/g56tr/.

d See the Supplementary material for a list of each article thesis.

e Dependent variables are rescaled from 0 to 1 for interpretability.

f Unlike prior research which has developed propaganda stimuli, we are unable to confirm the intended thesis of each article. Instead, unlike the determination for credibility, theses were identified and agreed upon for each randomly selected article by the authors prior to survey development.
