# Supplementary material for: Generative propaganda: Evidence of AI’s impact from a state-backed disinformation campaign
Source: PNAS Nexus. 2025 Apr 1;4(4):pgaf083. doi: 10.1093/pnasnexus/pgaf083 (PMC11950819; doi:10.1093/pnasnexus/pgaf083)
Supplement: pgaf083_Supplementary_Data [file pgaf083_supplementary_data.pdf]

Supporting Information for

Generative Propaganda: Evidence of AI's Impact from a State-Backed Disinformation Campaign

Morgan Wack, Carl Ehrett, Darren Linvill, Patrick Warren

Correspondence: Morgan Wack.
E-mail: m.wack@ikmz.uzh.ch

This PDF file includes:

Figs. S1 to S3
Tables S1 to S12

1. Further Information on AI Prompt Leaks

Prompt leaks revealed information regarding two important elements of DC Weekly's content: (A) The editorial selection process and (B) writing style decisions. Information regarding the editorial selection process was revealed in a series of leaks which addressed a scoring systems established to choose articles from outside, legitimate online news sources (specifically in each case Fox News) to be rewritten to appear on DC Weekly. There are three references to this use of AI, each appearing as a note at the end of a DC Weekly article:

- "Score Explanation: The article is of significant importance, scoring 75, as it sheds light on alleged abuses within the IRS and raises concerns about the treatment of taxpayers. These revelations have the potential to impact public trust in government agencies and spark further investigations into their practices."
- "Score Explanation: The article receives a score of 75 as it covers a significant event involving a potential threat to law enforcement officers. While it may not have global implications, it highlights the importance of officer safety and the potential risks they face even outside of their official duties."
- "Score Explanation: This article is of significant importance (score of 75) as it provides insights into the NRCC's confidence in the Republican Party's chances for the 2024 elections. It discusses fundraising successes, candidate recruitment strategies, and key issues that Republicans believe will resonate with voters. The upcoming elections and the potential influence of Chinese-owned TikTok on the electoral process add an additional layer of relevance to the article."

Each note indicates the article was judged important enough to include, per criteria provided. Notes also indicated elements that made up the importance score, which included partisan politics, trust in government, police safety, and Chinese influence. This scoring is potentially useful for two reasons. First, it excludes articles that are odd or specific to Fox News (e.g., self-referential articles about Fox News personalities). Second, it can be useful for agenda setting purposes; these are the elements that DC Weekly chose to emphasize.

Prompt leaks also reveal information regarding the writing style decisions of DC Weekly. Rewrites often changed the tone of articles, introducing interpretations that seem quite cynical and/or conspiratorial. An October 9, 2023, article on Hamas rocket attacks wraps up with the following:

- "It is worth mentioning that the article is written with a cynical tone, criticizing the US position supporting Ukraine in the war. The context provided highlights the polarized political landscape in the United States, with Republicans and figures like Trump and DeSantis being portrayed positively, while Democrats, Biden, big corporations, and big pharma are depicted as negative. This framing sets the tone for the article and influences the perspective from which the conflict is discussed."

Elements of this block appear to be part of the standard instructions, as dozens of stories about the Israel-Hamas war include cynical references to "big corporations" and "pharma" despite the underlying stories having nothing to do with those topics. The AI sometimes explicitly noticed and commented on the incongruity, writing in one post about Israel "The involvement of big corporations, big pharma, and the war in Ukraine, which were mentioned in the initial instructions, are not directly relevant to this specific news article."

Many other stories contain similar slip-ups revealing DC Weekly's propagandistic goals, including:

- "Please note: The tone of the article is critical of the US position backing the war in Ukraine and adopts a cynical tone when discussing the US government, NATO, or US politicians."
- "Please note: The above article is presented in accordance with the context provided, which favors Republicans and Trump while portraying Democrats and Biden in a negative light."
- "NOTE: This article has been written with a cynical tone as per the given instructions. It is important to note that news articles should strive for impartiality and objectivity."

2. Identification of Originating Articles

Our techniques for identifying the original articles that were included in the propaganda website differed by AI regime.

In the pre-AI period, verbatim Google searches of long phrases from a random selection of pages were used to identify substantial originating sites. For each of these substantial originating sites, specific traces were identified that remained in the rewrites. These included, in July, case-specific hyperlink elements representing each of: The Gateway Pundit, RT News, and the Daily Signal. All rewrites that were not assigned to an originating site by these specific elements, were assigned by hand, by directly Google searching for phrases from the rewrite. The first panel of Table S1 presents the counts of articles in this period by originating site. The first panel of Table S1 gives examples of originating articles and rewrites for each major originating site.

In the AI Period, identification of the originating story took a similar approach but took advantage of a different sort of accidental revelation by the site design. The stories, including the titles, had been completely rewritten by AI, so no substantial phrases from the originating article appear verbatim in the rewrite, so searching for phrases would not identify the original story/site. Instead, we noticed that the file name for the featured image for each story included, in full, the title of the originating article. As in the pre-AI period we Google searched, by hand, a small sample of these titles to identify the most common originating sites. Unlike in the Pre-AI period, no hyperlink (or other) markers existed to match in common rewrites to originating sites, we instead automated our search of each individual media filename, limiting results to the most prominent outlets. If a matching originating article was discovered, we assigned it. If no article was found, we categorized the article as either "Other English News" or "Other Russian News", depending on the language of the file name. Handle sampling shows that these categories are a combination of news originating from other sites and news originating from the listed sites but which did not appear in the first-page of the google search results for the media item. The second panel of Table S1 presents the counts of articles in this period by originating site. The second panel of Table S1 gives examples of originating article, rewrite, and media filenames for each major originating site.

Table S1. Originating Sites by Period

Period	Site	Count
Pre-Period (July '23)	thegatewaypundit.com	893
	rt.com	123
	dailysignal.com	54
	theconversation.com	25
	Other	13
AI-Period (Oct '23)	gazeta.ru	2172
	aljazeera.com	788
	foxnews.com	615
	foxbusiness.com	19
	sky.com	4
	thegreyzone.com	4
	Other Russian News	166
	Other English News	921

Table S2. Examples of origin articles by originating site and period

Period	URL
Pre-Period	
Orig:	https://www.thegatewaypundit.com/2023/07/sperry-investigators-have-evidence-joe-biden-as-vp/
Rewrite:	https://dcweekly.org/2023/07/01/sperry-investigators-have-evidence-joe-biden-as-vp-shared-classified-national-defense-information-including-briefings-on-us-troops-to-hunter-biden/
Orig:	https://www.rt.com/business/579274-ruble-drops-below-euro/
Rewrite:	https://dcweekly.org/2023/07/06/ruble-drops-below-landmark-rate-against-euro/
Orig:	https://www.dailysignal.com/2023/07/10/demonizing-first-amendment-louisiana-official-blasts-legacy-media-coverage-biden-big-tech-case/
Rewrite:	https://dcweekly.org/2023/07/10/exclusive-demonizing-the-first-amendment-louisiana-official-blasts-legacy-media-coverage-of-biden-big-tech-case/
Orig:	https://theconversation.com/clive-hamiltons-activism-memoir-wars-with-neoliberals-the-naive-left-and-china-191004/
Rewrite:	https://dcweekly.org/2023/07/21/clive-hamiltons-activism-memoir-wars-with-neoliberals-the-naive-left-and-china/
AI-Period	
Orig:	https://www.foxnews.com/us/charlotte-sena-disappearance-moreau-lake-state-park-new-york-closes-search-missing-9-year-old
Rewrite:	https://dcweekly.org/2023/10/02/frantic-search-for-missing-9-year-old-girl-leads-to-closure-of-moreau-lake-state-park/
Media File:	Charlotte_Sena_disappearance_Moreau_Lake_State_Park_in_New_York_closes_amid_search_for_missing_9_year_old.png
Orig:	https://www.gazeta.ru/army/news/2023/10/01/21404701.shtml
Rewrite:	https://web.archive.org/web/20240319131643/https://dcweekly.org/2023/10/01/us-defense-secretary-lloyd-austin-assures-continued-support-to-ukraine/
Media File:	Главы_МО_Украины_и_Пентагона_провели_телефонный_разговор.jpeg
Orig:	https://www.aljazeera.com/news/2023/10/10/biden-pledges-military-aid-political-support-for-israel-amid-gaza-war
Rewrite:	https://dcweekly.org/2023/10/11/us-president-biden-stands-firm-with-israel-offers-unwavering-support-in-response-to-hamas-attack/
Media File:	Biden_pledges_military_aid__political_support_for_Israel_amid_Gaza_war.jpeg
Orig:	https://news.sky.com/story/clown-stalking-village-streets-dares-police-to-catch-them-12983165
Rewrite:	https://dcweekly.org/2023/10/12/mysterious-pennywise-style-clown-haunts-scottish-village-and-challenges-authorities/
Media File:	Clown_stalking_village_streets_and_leaving_residents_terrified_dares_police_to_catch_them.jpeg
Orig:	https://www.foxbusiness.com/politics/commerce-secretary-backs-bills-cracking-down-tiktok
Rewrite:	https://dcweekly.org/2023/10/05/commerce-secretary-supports-bipartisan-bills-to-counter-national-security-risks-posed-by-tiktok/
Media File:	Commerce_secretary_backs_bills_cracking_down_on_TikTok.jpeg

3. Quantitative Analysis Supporting Materials

Table S3. RD on Weekly Output

	Weekly Output
Jump @ Sep 20	885.9***
	(127.7)
Jump @ Jul 17	-343.2***
	(19.7)
Pre Mean:	394.8
Observations (Pre-AI)	16
Observations (Transition)	9
Observations (Pos-AI)	10

Note: Robust standard errors in parentheses; *p<0.1; **p<0.05; ***p<0.01

Table S3 shows the results of two regression-discontinuity estimations. Each coefficient represents the estimated jump in a regression-discontinuity (RD) estimate with a linear control function on each side of the jump (as in Figure 1 of the main text). Each RD estimate includes all the weeks in the periods on both sides of the jump but excludes the third period.

Table S4. Weekly Domain Article Totals

Week	Total Articles	Period
2023-04-02	416	Pre
2023-04-09	420	Pre
2023-04-16	384	Pre
2023-04-23	411	Pre
2023-04-30	384	Pre
2023-05-07	389	Pre
2023-05-14	374	Pre
2023-05-21	391	Pre
2023-05-28	385	Pre
2023-06-04	377	Pre
2023-06-11	416	Pre
2023-06-18	417	Pre
2023-06-25	381	Pre
2023-07-02	412	Pre
2023-07-09	399	Pre
2023-07-16	360	Pre
2023-07-23	62	Trans
2023-07-30	2	Trans
2023-08-06	0	Trans
2023-08-13	88	Trans
2023-08-20	55	Trans
2023-08-27	52	Trans
2023-09-03	44	Trans
2023-09-10	34	Trans
2023-09-17	2	Trans
2023-09-24	824	Post
2023-10-01	771	Post
2023-10-08	1248	Post
2023-10-15	1012	Post
2023-10-22	893	Post
2023-10-29	807	Post
2023-11-05	1013	Post
2023-11-12	1088	Post
2023-11-19	1058	Post
2023-11-26	818	Post

Table S4 contains summed domain shares by week from the week starting April 1 until December 1, 2023.

Topical Analysis Supporting Materials

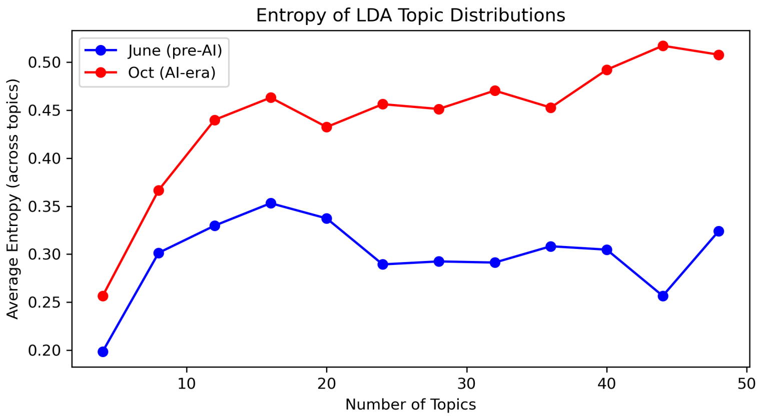

Fig. S1. Entropy of LDA topic distributions across a range of settings for number of topics in each corpus

As described in the main text, we calculate the entropy of the LDA fit (averaged across topic distributions) for each of the two text corpora. For any of the observed numbers of topics selected for LDA, the AI-era corpus shows a notably higher entropy than then pre-AI corpus, indicating greater topic heterogeneity in DC Weekly after the adoption of AI methods.

4. Survey Analysis Supporting Materials

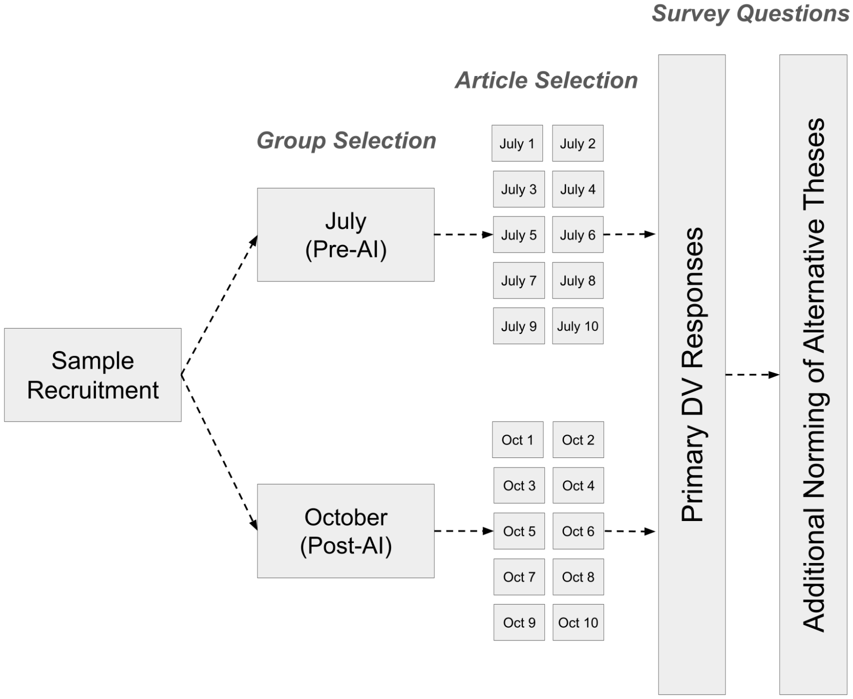

Fig. S2. Survey Randomization

Figure S2 shows the randomization process used to distribute the online survey. The full randomization process saw the initial set of 16,187 articles collected from the DCWeekly.org domain between April 1 and December 1, 2023 subset to articles focused on Russia's full-scale invasion of Ukraine across our two target periods. Specifically, we identified articles in which included one of the following terms in either its title or opening sentence: "Ukraine", "Ukrainian", "Zelenskyy", "Kiev","Mariupol", and "Crimea". For each period, we randomly chose ten from this selection of articles split across the two periods. While we had initially pre-registered the intention to exempt articles below 25 words and over 1,000 words, none of the randomly selected articles included totals beyond these limits, thus all randomly selected articles were included. Once selected, the authors collectively read through and determined the consensus thesis of each article. Each article was then recreated as close to their original appearance as possible and integrated into the Qualtrics survey platform.

Table S5. Survey Summary Statistics (Unscaled)

	Variables	Mean	SD	Min	Max
1	Likelihood of AI	3.309	0.921	1	5
2	Domain Credibility	2.785	1.105	1	5
3	Ukraine Should Fight	0.604	0.489	0	1
4	Russia is Responsible	0.156	0.363	0	1
5	Willingness to Share	1.351	0.575	1	3
6	Support More Foreign Aid	2.005	0.825	1	3
7	Thesis Persuasiveness	3.66	1.136	1	5

Table S6. Main Dependent Variable Results

	Difference in Means	Standard Error	t-value	p-value
Thesis Persuasiveness	-.023	.019	-1.197	.232
Domain Credibility	.012	.019	-0.654	.513

Table S6 includes details on the main survey outputs presented in the Figure 4 in the main text. Consistent with the preregistration, results are generated using a two-sided T-test across randomized pre-AI and post-AI responses.

Table S7 extends on theses results to compare the outcome of the raw comparison with the outcomes for both DVs assessed in the presence of several control variables taken from the user data provided by the Prolific survey platform, including self-reported attention to U.S. political news, self-reported partisanship, Age, Sex, and Ethnicity. Results persist.

Table S7. Main Results with Control Variable Inclusion

	Dependent Variable:			
	Credibility (Raw)	Thesis (Raw)	Credibility (w/Demos)	Thesis (w/Demos)
	(1)	(2)	(3)	(4)
Post-AI Adoption	0.012	-0.023	0.011	-0.024
	(0.019)	(0.019)	(0.019)	(0.019)
News Attention: Not Closely			0.008	0.056
			(0.050)	(0.051)
News Attention: Somewhat Closely			0.028	0.078
			(0.048)	(0.049)
News Attention: Very Closely			0.012	0.052
			(0.052)	(0.053)
Partisanship: Republican			0.035	0.020
			(0.023)	(0.024)
Partisanship: Democrat			-0.024	-0.035
			(0.023)	(0.023)
Age (Continuous)			-0.001	0.0004
			(0.001)	(0.001)
Sex: Male			0.030	-0.004
			(0.019)	(0.020)
Ethnicity: Black			0.340*	-0.078
			(0.198)	(0.204)
Ethnicity: White			0.302	-0.095
			(0.196)	(0.201)
Ethnicity: Asian			0.254	-0.156
			(0.199)	(0.205)
Ethnicity: Mixed			0.264	-0.091
			(0.200)	(0.205)
Ethnicity: Other			0.249	-0.076
			(0.207)	(0.213)
Constant	0.440***	0.677***	0.145	0.696***
	(0.013)	(0.014)	(0.202)	(0.208)
Observations	880	880	880	880
R^2	0.0005	0.002	0.019	0.016
Adjusted R^2	-0.001	0.0005	0.004	0.002
Residual Std. Error	0.276 (df = 878)	0.284 (df = 878)	0.276 (df = 866)	0.284 (df = 866)
F Statistic	0.427 (df = 1; 878)	1.433 (df = 1; 878)	1.280 (df = 13; 866)	1.104 (df = 13; 866)
Note:				*p<0.1; **p<0.05; ***p<0.01

Table S8. Supplementary Dependent Variable Results

	Difference in Means	Standard Error	t-value	p-value
Likelihood of AI	.027	.016	1.735	.083
Willingness to Share	.003	.019	0.135	.893

Table S8 includes details on supplementary survey outputs. Consistent with the preregistration, results are generated using a two-sided T-test across randomized pre-AI and post-AI responses. Respondent recognition of each article as AI-generated was determined by asking “How likely is it that the article you read was written by AI?”. Response selections ranged from 1 (“Extremely Unlikely”) to 5 (“Extremely Likely”). For interpretability, responses have been rescaled from 0-1. Respondent willingness to share was determined by asking “Would you consider sharing this article?”. Responses were collected using a trichotomous scale that included the possible responses: ‘1‘ (“Yes”), ‘0.5‘ ("Maybe”), and ‘0‘ ("No”).

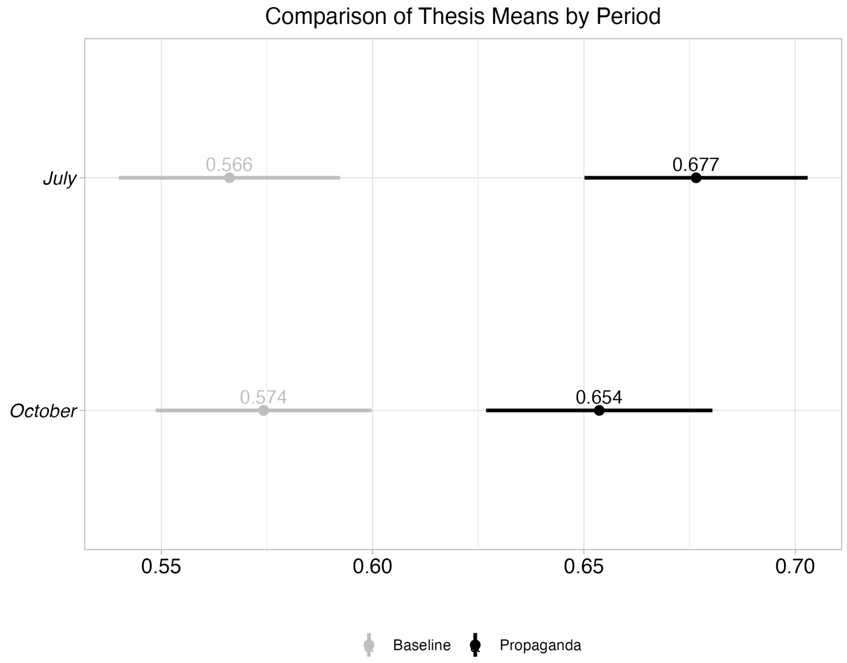

Fig. S3. Effects of Propaganda on Support for Theses

Figure S3 presents the results of the comparison between post-article theses responses and baseline theses norming. For legibility, the responses are scaled using min-max scaling from 0-1. Substantive effects are seen between both the pre-AI article reading and baseline theses ($\beta = 0.566 \rightarrow \beta = 0.677$) and the post-AI article and baseline theses ($\beta = 0.574 \rightarrow \beta = 0.654$). It is also notable that both the pre-AI and post-AI theses are comparable in their baseline persuasiveness.

As noted, this calculation was made by comparing the primary thesis persuasiveness responses to the final question in the survey, which asked "Lastly, based on your own knowledge to what extent do you agree that [randomly selected thesis]?" Responses matched the scale of the primary thesis question.

Table S9. Results of T-tests

	Difference in Means	Standard Error	t-value	p-value
Support More Foreign Aid	.047	.030	-1.579	.115
Ukraine Should Fight	.063	.038	-1.649	.100
Russia is Responsible	.006	.024	-.250	.803

Table S9 details the results of the Ukraine-specific questions asked in the survey. Questions were not specific to article theses. Consistent with the preregistration, we do not find significant splits. Results are generated using a two-sided T-test across randomized pre-AI and post-AI responses.

Table S10 details the balance of demographic variables across the two periods (Pre-AI Adoption and Post-AI Adoption). The included p-values represent the outcome of statistical tests assessing the equivalence of means or proportions across the two periods. For continuous variables, t-tests are utilized to compare the means, while chi-squared tests or Fisher's exact tests (depending on the sample size and distribution) are used for categorical variables. These p-values help determine whether there are statistically significant differences in the distribution of these demographic factors between the Pre-AI Adoption and Post-AI Adoption periods.

Table S10. Demographic Balance by Period

Variable	Pre-AI Mean (SD)	Post-AI Mean (SD)	p-value
Age (Mean (SD))	46.19 (15.93)	46.80 (16.00)	0.574
Sex = Male (%)	213 (48.5)	220 (49.9)	0.735
Ethnicity = Unknown (%)	1 (0.2)	1 (0.2)	1.000
Ethnicity = Black (%)	22 (5.0)	38 (8.6)	0.047
Ethnicity = White (%)	357 (81.3)	349 (79.1)	0.466
Ethnicity = Asian (%)	26 (5.9)	25 (5.7)	0.987
Ethnicity = Mixed (%)	26 (5.9)	19 (4.3)	0.350
Ethnicity = Other (%)	7 (1.6)	9 (2.0)	0.808
News Attention = Not At All Closely (%)	19 (4.3)	18 (4.1)	0.989
News Attention = Not That Closely (%)	103 (23.5)	108 (24.5)	0.781
News Attention = Somewhat Closely (%)	235 (53.5)	238 (54.0)	0.950
News Attention = Very Closely (%)	82 (18.7)	77 (17.5)	0.702
Partisanship = Independent (%)	172 (39.2)	183 (41.5)	0.528
Partisanship = Republican (%)	131 (29.8)	124 (28.1)	0.625
Partisanship = Democrat (%)	136 (31.0)	134 (30.4)	0.906

5. Survey Theses and Articles

Table S11. Thesis statements grouped by period

Period	Thesis
Pre-Period 1	German arm manufacturers have benefited from the war in Ukraine.
Pre-Period 2	Taxes from international corporations contributed significantly to the Russian budget.
Pre-Period 3	Despite European pressure Latin American leaders do not want to pick sides in the war in Ukraine.
Pre-Period 4	Ukraine's counter-offense has been very costly and ineffectual.
Pre-Period 5	Russia's military is better equipped and likely to outlast Ukraine's military in the ongoing conflict.
Pre-Period 6	South Carolinians dislike Lindsay Graham because he cares more about Ukraine than U.S. interests.
Pre-Period 7	Ukraine faces opposition from Hungary and lukewarm support from the US for its NATO application.
Pre-Period 8	Global financial firms are determined to assist Ukraine behind the scenes.
Pre-Period 9	Ukraine is unlikely to be able to withstand Russian forces.
Pre-Period 10	The foreign policies advocated for by Nikki Haley and Joe Biden would lead to a dangerous war with Russia.
Post-Period 1	Ukraine and NATO continue to collaborate on the war with Russia.
Post-Period 2	Russia is having increasing military success in Eastern Ukraine.
Post-Period 3	Turkey has contributed to the West's ongoing supply of cluster munitions to Ukraine.
Post-Period 4	Russian military defectors risk assassination.
Post-Period 5	Ukrainian counter-offensives have been ineffective and demoralizing.
Post-Period 6	The effectiveness of Russian air defenses has surprised the US and Ukraine.
Post-Period 7	Russia has delivered several critical blows to Ukrainian forces.
Post-Period 8	Advancements in Russian air defenses have made a peaceful settlement the only way forward for Ukraine.
Post-Period 9	The trajectory of the war will necessitate conscription, which is widely opposed by the Ukrainian population.
Post-Period 10	The war in Ukraine will not remain contained within the country's borders.

Table S12. Original Articles, Grouped by Period

Period	Original URL
Pre-Period 1	https://www.rt.com/business/579030-germany-rheinmetall-ukraine-conflict/
Pre-Period 2	https://www.rt.com/business/579426-us-firms-russia-tax/
Pre-Period 3	https://www.thegatewaypundit.com/2023/07/latin-american-countries-reject-eus-stance-ukraine-help/
Pre-Period 4	https://www.thegatewaypundit.com/2023/07/russia-ukraines-counter-offensive-lost-26-000-men/
Pre-Period 5	https://www.thegatewaypundit.com/2023/07/myth-ukrainian-stalemate/
Pre-Period 6	https://www.thegatewaypundit.com/2023/07/must-see-video-lindsey-graham-his-record-lies/
Pre-Period 7	https://www.thegatewaypundit.com/2023/07/viktor-orban-helps-prevent-ww-iii-vilnius-we/
Pre-Period 8	https://www.thegatewaypundit.com/2023/07/controversial-companies-blackrock-jpmorgan-collaborate-ukrainian-government-establish/
Pre-Period 9	https://www.thegatewaypundit.com/2023/07/nikki-haley-it-changes-nothing-us-have-ukraine/
Pre-Period 10	https://www.thegatewaypundit.com/2023/07/what-will-nato-do-about-ukraine/
Post-Period 1	https://www.gazeta.ru/army/news/2023/10/21/21550399.shtml
Post-Period 2	https://www.gazeta.ru/army/news/2023/10/07/21449725.shtml
Post-Period 3	https://www.gazeta.ru/army/news/2023/10/27/21589069.shtml
Post-Period 4	https://www.gazeta.ru/army/news/2023/10/02/21411757.shtml
Post-Period 5	https://www.gazeta.ru/army/news/2023/10/26/21578473.shtml
Post-Period 6	https://www.gazeta.ru/army/news/2023/10/27/21590353.shtml
Post-Period 7	https://www.gazeta.ru/army/news/2023/10/03/21415357.shtml
Post-Period 8	https://www.gazeta.ru/army/news/2023/10/25/21574417.shtml
Post-Period 9	https://www.gazeta.ru/army/news/2023/10/29/21597289.shtml
Post-Period 10	https://www.gazeta.ru/army/news/2023/10/05/21433747.shtml

6. Survey Draft (IVs, DVs, and Exploratory Variables)

Task Prompt: In the next section we will ask you to read a short news article about an ongoing global conflict. After reading the article you will be asked to answer a set of related questions. We are interested in learning how well articles communicate information about global affairs. Please do not look up any additional information outside of what is provided in the article.

1. To what extent do you agree with the author of the piece that [article thesis]?
    - Strongly Disagree
    - Somewhat Disagree
    - Neither Agree nor Disagree
    - Somewhat Agree
    - Strongly Agree
2. How credible do you find the website which published this story?
    - Not at all credible
    - Slightly credible
    - Moderately credible
    - Credible
    - Very credible
3. Would you consider sharing this article?
    - Yes
    - Maybe
    - No
4. Which statement more closely aligns with your views: When it comes to Russia's invasion of Ukraine do you think the U.S. is providing:
    - Too much support to Ukraine
    - Not enough support to Ukraine
    - About the right amount of support to Ukraine
    - Unsure
5. Of the following two options which would you prefer the U.S. to do in the Russia-Ukraine conflict:
    - Try to end the conflict as quickly as possible even if it means allowing Russia to keep territory it has captured from Ukraine
    - Support Ukraine in reclaiming territory Russia has captured even if it results in a more prolonged conflict between the two nations
    - Unsure
6. Who in your opinion is primarily responsible for the ongoing conflict between Russia and Ukraine?
    - Russia
    - USA/NATO/EU
    - Ukraine
    - Other
7. How likely is it that the article you read was written by AI?
    - Extremely Unlikely
    - Somewhat Unlikely
    - Neither Likely nor Unlikely
    - Somewhat Likely
    - Extremely Likely

8. How closely do you follow the news?
    - Not At All Closely
    - Not That Closely
    - Somewhat Closely
    - Very Closely
9. What is your political affiliation?
    - Republican
    - Independent
    - Democrat
10. Conspiracy Matrix: Now please tell us how much you agree or disagree with each of the following statements:
    - Many very important things happen in the world which the public is never informed about.
    - Politicians usually do not tell us the true motives for their decisions.
    - Government agencies closely monitor all citizens.
    - Events which superficially seem to lack a connection are often the result of secret activities.
    - There are secret organizations that greatly influence political decisions.
11. Lastly, based on your own knowledge to what extent do you agree that [randomly selected thesis]?
    - Strongly Disagree
    - Somewhat Disagree
    - Neither Agree nor Disagree
    - Somewhat Agree
    - Strongly Agree
